# The impact of inherently aversive contexts on visuocortical processing of generalized threat

**DOI:** 10.1162/IMAG.a.74

**Published:** 2025-07-07

**Authors:** Yannik Stegmann, Matthias Gamer

**Affiliations:** Department of Psychology (Experimental Clinical Psychology), University of Würzburg, Würzburg, Germany

**Keywords:** EEG, threat, generalization, SSVEP, context, attention, learning

## Abstract

Adapting behavior to environmental demands is a fundamental aspect of survival. In the face of unfamiliar potential dangers, organisms display a wide range of defensive mechanisms, such as using contextual information to prepare for upcoming threats and extrapolating from previous experiences with similar encounters (threat generalization). Importantly, these different types of threat-related information place distinct demands on the attentional system: potential, context-related threat induces a state of hypervigilance, whereas imminent, acute threat requires selective attention. While these individual mechanisms are increasingly well understood, their interactions remain elusive, particularly at the neurophysiological level. Therefore, the current study aimed to orthogonally combine threat generalization with aversive contextual information and measure correlates of defensive behavior on a subjective, autonomic, and electrocortical level. Fifty-two human participants completed a threat generalization paradigm followed by a context phase in which the conditioned visual cues were presented against aversive or neutral background images, respectively. Results revealed successful threat generalization for subjective and pupillary responses with overall heightened responses for cues presented in aversive compared to neutral contexts. For visuocortical activity as measured by steady-state visually evoked potentials (ssVEPs), this response pattern was separated into different frequencies. While the fundamental frequency showed the general main effect of aversive contexts, the second harmonic followed a generalization gradient, suggesting a segregation of competing attentional demands via neural harmonics. Together, these findings provide new insights into adaptive defensive behavior in complex situations, characterized by an additive model of different defensive processes.

## 1 Introduction

Adapting behaviors to meet environmental demands is crucial for survival in threatening situations. Organisms have developed specialized processes to ensure appropriate responses to potential threats, as each encounter carries the possibility of a fatal outcome (Fanselow, 2018). To navigate these dangers, organisms rely on a wealth of information gathered from their surroundings. When confronted with a novel, potentially threatening stimulus, organisms respond based on their experiences with similar stimuli in the past. This phenomenon, where learned threat extends to a new stimulus or situation, is known as aversive generalization (Dunsmoor et al., 2009; Lissek et al., 2008). The ability to generalize threat is a vital survival mechanism, allowing organisms to prepare for and respond to threats that share characteristics with previous dangers (Dunsmoor & Paz, 2015). To test models of aversive generalization in the laboratory, individuals are usually presented with a stimulus (CS+) that is paired with an aversive outcome (unconditioned stimulus, US), such as a mildly painful electrical stimulation. Then, additional stimuli (generalization stimuli, GS) are presented that share some similarity with the original threat-signaling stimulus but have never been directly associated with the US (Dunsmoor & Paz, 2015). Activation of defensive mechanisms in response to these GS increases with the degree of similarity to the CS+ as indexed by somato-visceral measures such as skin conductance (Dunsmoor et al., 2009), heart rate (Ahrens et al., 2016), and fear-potentiated startle (Lissek et al., 2008), as well as subjective ratings of perceived threat (Stegmann, Schiele, et al., 2019). Successful aversive generalization has already been demonstrated for stimuli sharing perceptual similarities in low-level features such as color (Dunsmoor & LaBar, 2013), physical size (Lissek et al., 2008), and orientation (McTeague et al., 2015). However, these processes do not seem to depend on such isolated perceptual features as similar results were also reported for more complex stimuli, including categories (Dunsmoor & Murphy, 2014), faces (Reutter & Gamer, 2023), and contexts (Andreatta et al., 2015). Aversive generalization is often interpreted within a “better safe than sorry” framework (Dunsmoor & Paz, 2015), suggesting that from an evolutionary perspective, the increased chances of survival outweighed the costs of potential false alarms. However, an overgeneralization of learned threat can result in excessive avoidance behaviors, which are closely related to pathological forms of anxiety (Cooper et al., 2022; Fraunfelter et al., 2022).

To benefit from the generalization memory in acute situations, the organism is required to have identified an imminent threat, which, in many cases, may occur too late to allow for effective preventive action. Thus, even before encountering an actual threat, organisms utilize information from the environment to prepare for defensive actions. Indeed, contextual factors play an important role in shaping defensive responses to upcoming, distal threats (Fanselow & Lester, 1988; Mobbs et al., 2015). When entering a context that is inherently aversive or has been linked to threat before, organisms engage in a state of sustained defense system activation (Davis et al., 2010), which is reflected in heightened levels of electrodermal activity and heart rate bradycardia (Lang et al., 2000). Furthermore, attentional processes are characterized by hypervigilance, aimed at enhancing the detection of potential threats (Richards et al., 2014). Together, these processes have been suggested to strengthen defensive responses once an actual threat has been identified. However, the effect of aversive contextual information on attentional engagement toward an actual threat remains less clear, as it imposes two potentially competing demands on the attentional system. While imminent threat typically narrows attention, focusing it on the threatening stimulus (also known as selective attention), hypervigilance induced by potential contextual threat broadens the attentional ‘spotlight’ and has been associated with increased environmental scanning (Richards et al., 2014). This raises the question of whether the presence of contextual threat enhances attentional engagement toward the immediate threat cue, amplifying selective attention, or whether it diffuses attentional resources, leading to a more distributed allocation of attention across both cue and context. To test this hypothesis, we previously employed an orthogonal cue in context conditioning paradigm, in which conditioned stimuli were presented in front of inherently aversive or neutral background pictures (Stegmann et al., 2022, 2024). Indeed, we demonstrated that aversive contexts engaged the defense system, as indicated by sustained heart rate deceleration. More importantly, conditioned threat stimuli were perceived as more threatening and elicited stronger skin conductance and heart rate responses when encountered in an aversive compared to a neutral context. Additionally, consistent with theories linking heart rate deceleration to enhanced sensory processing and heightened vigilance, we observed stronger attentional responses as measured by visuocortical activity during CS+ presentations in aversive compared to neutral contexts. To achieve this, we leveraged the benefits of the steady-state visual evoked potential (ssVEP), an electrocortical response to flickering stimuli that can be readily quantified in the EEG (Norcia et al., 2015). The frequency of the ssVEP matches the driving frequency of the stimulus, resulting in an excellent signal-to-noise ratio (Regan, 1989). Spatiotemporal analysis of the ssVEP has identified the extended visual cortex, including V1 and higher-order visual regions, as likely cortical sources (Di Russo et al., 2007; Muller et al., 1997), highlighting its connection to both sensory processing and attentional mechanisms. The amplitude of the ssVEP is sensitive to top-down modulations by higher-order processes, such as spatial attention (Muller et al., 1998), working memory (Silberstein et al., 2001), as well as emotional arousal (Keil et al., 2003). Furthermore, a vast body of aversive conditioning studies was able to demonstrate enhanced ssVEP amplitudes to CS+ compared to safety stimuli (Miskovic & Keil, 2012). In line with the general notion of heightened attentional processing of potentially threatening stimuli during states of sustained defense system activation, it is assumed that these adaptations in the visuocortical system facilitate improved detection of threat-relevant cues (Rhodes et al., 2018; Stegmann et al., 2021). Supporting this idea, recent studies have demonstrated not only an enhanced gain for threat-relevant stimuli but also a concurrent inhibition of threat-irrelevant stimuli, reflecting optimized discrimination between threat-relevant and -irrelevant features (Moratti et al. 2017; Santos-Mayo et al., 2022). Additionally, ssVEPs can be used with overlapping stimuli flickering at different frequencies to quantify the visuocortical responses to each cue via frequency tagging (Wieser & Keil, 2011, 2014), making them well suited for cue-in-context paradigms. For example, a recent study by Santos-Mayo and Moratti (2025) employed a differential threat learning paradigm with a flickering neutral context cue in the background. The authors found that visuocortical engagement with the context cue increased linearly throughout the acquisition phase when no CS was present, but this engagement diminished when a CS was presented. These findings suggest a complex interplay between cue and background processing, likely reflecting dynamic shifts in attentional demands. However, the mechanisms by which contextual information, especially aversive contexts, affects attentional processing of threatening cues remain elusive. For conditioned fear responses in general, previous studies have reported mixed results, with some findings supporting an additive model and others supporting an interactive model of contextual modulation (Bublatzky et al., 2010; Grillon & Charney, 2011; Somerville et al., 2013; Stegmann et al., 2022, 2024). The main difference between the additive and interactive models lies in how aversive contexts influence defensive responses to safety and threat cues (Stegmann et al., 2022). The additive model predicts that aversive contexts amplify overall defensive behavior, regardless of the type of cue. In contrast, the interactive model suggests that aversive contexts selectively enhance defensive responses to threat cues, rather than to both safety and threat cues.

A promising approach to further disentangle additive and interactive mechanisms is to explore how aversive contexts modulate aversive generalization processes. While defensive responses usually increase with greater similarity to the CS+, ssVEP studies have sometimes shown a pattern characterized by heightened responses to the CS+ whereas the responses to the CSs, that are most similar to the CS+, are suppressed (Antov et al., 2020; McTeague et al., 2015). Such a pattern is consistent with models of attentional competition (Santos-Mayo & Moratti, 2025) and can be explained by mechanisms of lateral inhibition between neighboring feature-selective neurons. It has been observed in neuronal populations sensitive to orientation (Antov et al., 2020; McTeague et al., 2015), spatial location (Friedl & Keil, 2021), and higher-order visual stimuli such as faces (Pouliot et al., 2025; Stegmann et al., 2020). It is assumed that this pattern of sharpened re-tuning in neurons within the visual cortex reflects enhanced perceptual discrimination as a result of aversive learning (Pouliot et al., 2025).

The present study sought to extend these findings to cue–context interactions, investigating whether ssVEP responses in aversive contexts conform to an additive or interactive model. Similarly to previous studies (Righart & de Gelder, 2005, 2008; Stegmann et al., 2022; Wieser & Keil, 2014), we used IAPS pictures (Lang et al., 2008) as inherently affective backgrounds to induce aversive states. An additive model would manifest as overall increased ssVEP amplitudes in response to cues presented in aversive compared to neutral contexts, while the shape of the generalization gradient remains stable. An interactive model, however, would be characterized by differences in the shape of generalization gradients between contexts. This could result in either a widening (indicating overgeneralization) or sharpening (indicating enhanced discrimination) of the generalization pattern. To substantiate visuocortical responses with indices of sympathetic nervous system activity, we also measured pupillary responses to the central cues as a function of context. For changes in pupil diameter, we expected enhanced responses to the CS+, accompanied by a decreasing gradient with increasing dissimilarity to the CS+ (Reutter & Gamer, 2023). This gradient would be either generally higher (additive model) or more/less wide (interactive model) in the aversive compared to the neutral context.

## 2 Method

### 2.1 Subjects

A total of 52 participants (44 female; age: *M* = 23.00, *SD* = 3.66 years) completed the study. Prior to participation, written informed consent was obtained from each participant. The study and all protocols were pre-registered (see https://osf.io/a27yh) and approved by the ethics review board of the University of Würzburg. Participants were recruited via a local platform (https://psywue.sona-system.com) and were at least 18 years old, had normal or corrected-to-normal vision, no past or present psychiatric diagnosis (self-report), and no current pregnancy. All participants were paid 12 € per hour or received course credit for participation. Data from all participants who completed the study were used for analysis of the pupillary responses and ratings. For the analysis of the EEG data, 4 participants were excluded due to excessive noise and recording failure, leaving 48 participants (41 female; age: *M* = 23.13, *SD* = 3.73 years).

The planned sample size of n ~ 50 was derived from a power simulation conducted for our previous study (Stegmann et al., 2022), for which we assumed medium effect sizes (*d* = .30) for the effect of aversive contexts on central cue responses. For simulating repeated measures data, standard deviations (*SD* = 1.4) and correlations between measures (*rho* = 0.80) were estimated by aggregating our previous data. A simulation of 5000 tests detected a significant main effect of context in at least 80% of the simulated tests for n = 50 participants at an alpha level of 5%.

### 2.2 Stimuli and apparatus

Conditioned stimuli (CS) consisted of circular black-and-white sinusoidal grating stimuli (spatial frequency: 0.1 cycles per pixel) filtered with a Gaussian-envelope (i.e., Gabor-patch) with maximum contrast of 100% at center (Stegmann et al., 2022). Identical to the study of McTeague et al. (2015), the orientation of 45° relative to the vertical axis was selected as CS+ cue, while the orientations of -45°, +15°, +25°, +35°, +55°, +65°, and +75° served as generalization stimuli (all CS-). All stimuli were presented on a gray background at the center of a 17-inch monitor (resolution = 1280 x 1024 pixel). From a viewing distance of 80 cm the conditioned stimuli spanned approximately 7.2° of visual angle horizontally and vertically. To elicit ssVEPs, all CS were presented in a flickering mode using a square wave luminance modulation at a frequency of 7.5 Hz (with equal on-off cycles).

Similar to our previous study (Stegmann et al., 2024), six aversive (CTX_a_) and six neutral pictures (CTX_n_) from the IAPS (Lang et al., 2008) were selected as context stimuli according to their normative ratings of valence (mean unpleasantness on a 9-point scale with 9 indicating maximum unpleasantness: aversive: 8.14; neutral: 4.64) and arousal (mean arousal on a 9-point scale with 9 indicating highest arousal: aversive: 6.01; neutral: 3.44). The picture categories contained an equal number of social and animal content (catalog numbers of the IAPS pictures used in this study are as follows: aversive, 3015, 3064, 6520, 9181, 9185, 9254; neutral, 1350, 1670, 2026, 2036, 2235, 2393) and were converted to grayscale, with average luminance matched to the gray background. Each picture was presented only once during the experiment, without flickering, and spanning visual angles of 13.90° horizontally and 10.36° vertically. At the end of the experiment, participants rated the valence (ranging from 1 = “very pleasant” to 9 = “very unpleasant”) and arousal (ranging from 1 = “very calm” to 9 = “very arousing”) of the IAPS background pictures using a computer-based version of the Self-Assessment Manikin Scale (Bradley & Lang, 1994) to ensure that they perceived the aversive or neutral pictures as such. Results confirmed higher unpleasantness (aversive vs. neutral: 6.61 vs. 3.07, *t*(51) = 24.59, *p* < .001, *d* = 3.41, *CI_95_* = [2.69; 4.12]) and arousal (aversive vs. neutral: 5.74 vs. 1.98, *t*(51) = 16.31, *p* < .001, *d* = 2.26, *CI_95_* = [1.74; 2.77]) for aversive compared to neutral pictures.

The US consisted of a 50 ms electrical pulse train (2 ms pulse width, separated by 4 ms), which was delivered by a constant current stimulator (Digitimer DS7A, Digitmer Ltd., Welwyn Garden City, UK) to the left forearm through a surface bar electrode consisting of two stainless-steel disks of 9 mm diameter and 30 mm spacing. US intensities were adjusted to the individual pain threshold, using a staircase-procedure consisting of two ascending and descending series of electrical stimuli. After each stimulation, participants rated their sensation on a scale from 0 = “not painful at all” to 10 = “very painful”, with 4 indicating “just noticeable pain”. The procedure began with a stimulation intensity of 0.25 mA, which was increased in 0.25 mA increments until participants reported a sensation greater than 6. From this point, the intensity was decreased in steps until the rating dropped below 6, after which the next ascending series began targeting a reported sensation of 7. In the final descending series, stimulation was terminated once a rating of exactly 6 was reached, and this intensity was defined as the individual US level. After calibration, participants were asked to rate the US unpleasantness of the final intensity again, resulting in a mean US unpleasantness of 5.74 ± .72 (*M* ± *SD*) and a mean US intensity of 1.98 ± 1.73 mA. Note that the final score for US unpleasantness could be below 6 as every final rating above 4 after the calibration was considered acceptable.

### 2.3 Design and procedure

**Fig. 1.**
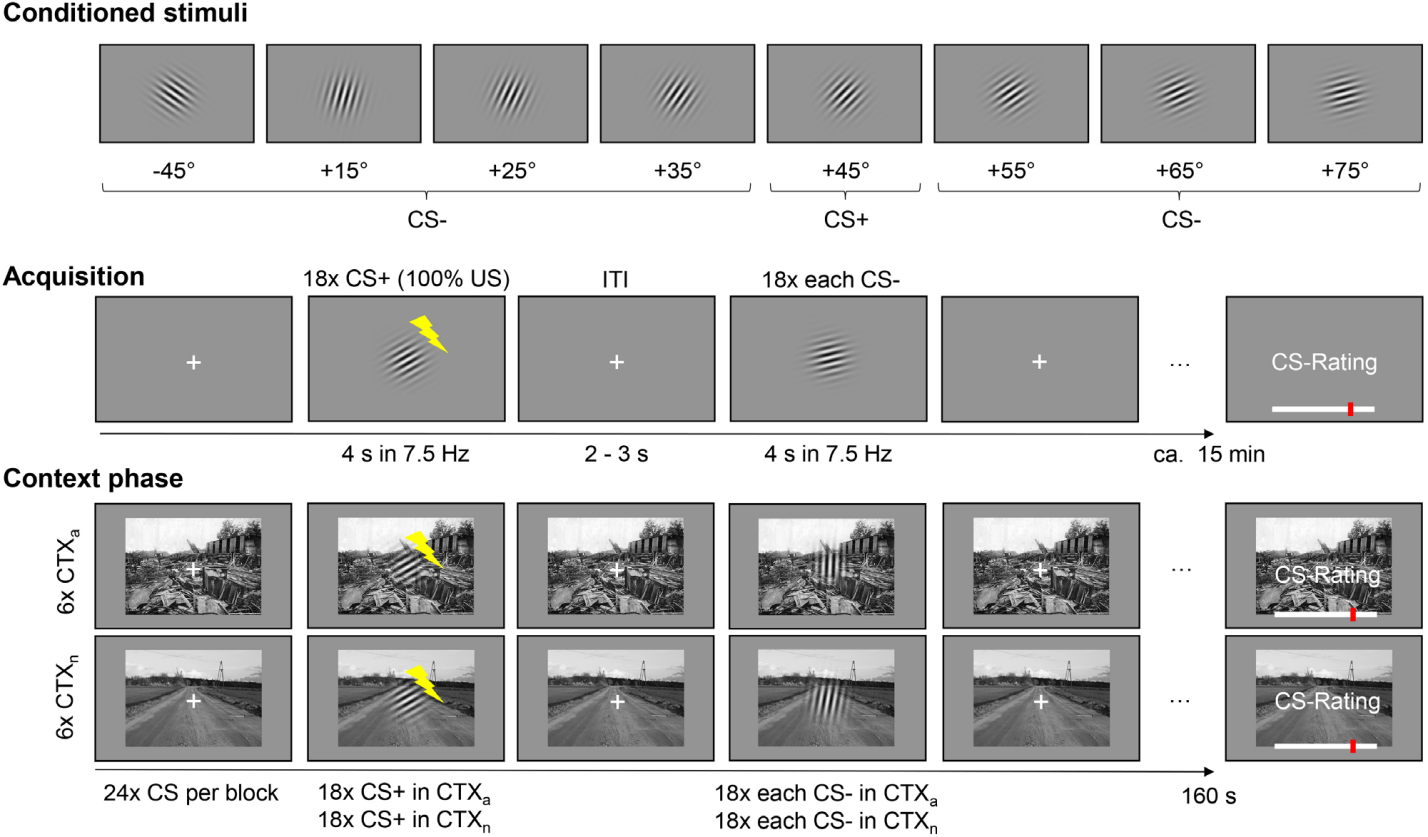
Stimuli and design. During the acquisition phase, the CS+ and each of the seven differently oriented CS- (top row) were presented 18 times for 4 s, using a 7.5 Hz flicker mode (on-off flicker). In the context phase, six blocks of aversive (CTX_a_) and neutral (CTX_n_) contexts were used. Each block began with the presentation of a background picture, followed by 24 CS presentations with the same timing and reinforcement rate (illustrated as yellow lightnings) as during acquisition, resulting in a total of 18 presentations per orientation and context condition. Context-dependent ratings of the CS were collected after each block, with the background pictures remaining on screen. Note that the background images shown in this figure are examples and differ from those used in the original experiment.

After obtaining written informed consent and completing the sociodemographic questionnaire, the stimulation electrode was applied to the participants, and they completed the shock work-up procedure. The main paradigm consisted of an acquisition phase, which was followed by the cue in context phase (see Fig. 1). During the acquisition phase, 18 trials of CS+ (+45° orientation) and each CS- orientation (-45°, +15°, +25°, +35°, +55°, +65°, +75°) were presented for 4 s. The US co-terminated with the CS+ orientation in 100% of the trials and subjects were not informed of any specific relation among the CSs and the US. Trial order was pseudo-randomized so that no more than three identical CSs could occur consecutively. CS presentations were separated by an ISI of 2–3 s. After acquisition, subjects were asked to rate US expectancy (“What is the likelihood that the currently presented stimulus is followed by an electrical stimulus?”; from 0% = “not likely” to 100% = “very likely”) for each orientation via electronical visual analog scales.

The context phase consisted of six blocks of aversive (CTX_a_) and six blocks of neutral (CTX_n_) contexts. Each block began with the presentation of either an aversive or neutral background picture, and the first CS was presented after 5–7 s relative to background picture onset. During each block, 24 CS were presented using the same timing and reinforcement rate as during acquisition, resulting in a total of 18 presentations per orientation and context condition. The background pictures remained on screen throughout the block, which lasted approximately 160 s. After each block, context-dependent US shock expectancy ratings of the CS were obtained while the background pictures remained on screen.

### 2.4 Physiological data recording and processing

Pupil diameter was registered via a Tobii Pro Nano eye tracker at a sampling frequency of 60 Hz. Missing data due to eye blinks or loss of tracking were linearly interpolated, before filtering the average pupil diameter of both eyes with a 2 Hz low-pass filter. Changes in pupil diameter were then calculated by subtracting the baseline mean of a time window from -500 to 0 ms relative to cue onset from each ensuing datapoint. For the statistical analysis, we calculated the average changes in pupil diameter within 500 to 4000 ms relative to cue onset for every condition (Reutter & Gamer, 2023).

**Fig. 2.**
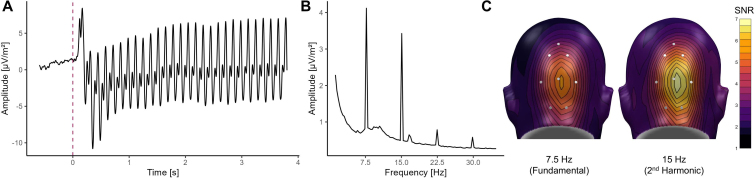
(A) Grand average of the EEG signal at sensor Oz during the context phase in the time domain, illustrating a distinct steady-state visual evoked potential to the onset of the central cue (red dashed line). (B) The frequency spectrum of the grand average confirms amplitude peaks at the driving frequency 7.5 Hz and its harmonics, with a particularly strong amplitude at the second harmonic (15 Hz). (C) Topographies of the grand average 7.5 Hz and 15 Hz signal-to-noise ratios show maximal visuocortical activity over occipital sensors. The white dots illustrate the sensors used for statistical analysis.

The EEG was continuously recorded via 129 electrodes using an Electrical Geodesics (EGI, Eugene, OR, USA) high-density EEG System referenced to Cz, with a sampling rate of 500 Hz and online bandpass filtered with 0.1 and 100 Hz and a 50 Hz notch filter. Impedances were kept below 50 kΩ as recommended for the Electrical Geodesics high-impedance amplifiers. Epochs of 600 ms pre-stimulus and 3900 ms post-stimulus onset were extracted using the software EMEGS (Electro Magnetic Encephalography) for Matlab (Peyk et al., 2011). The last 100 ms of the cue presentation were discarded to exclude potential effects of the co-terminating US presentations. Data were then filtered with a 40 Hz low-pass filter (45 dB/octave, 23rd-order Butterworth). In a next step, we used the SCADS procedure (Junghöfer et al., 2000) for artifact handling. Trials with artifacts were identified based on the distribution of statistical parameters (absolute value, standard deviation, and maximum of the differences) of the trials and sensors. Contaminated sensors were replaced by statistically weighted, spherical spline interpolated values. However, trials were rejected completely when more than 20 out of 129 sensors were contaminated. On average, we excluded 30.6 % of trials from the analysis (*SD* = 13.2 %). Artifact-free trials were then averaged separately for each subject and experimental condition. For the analysis of learning dynamics during the acquisition phase, trials were further divided into the first and second halves of the acquisition phase, resulting in the additional exclusion of n = 7 participants due empty cells in at least one of the Cue × Time (1^st^ vs. 2^nd^ half) conditions. Note that these participants were only excluded for the analysis of ssVEPs during the acquisition phase. To reduce topographical variability between subjects, we calculated the current source densities (CSD) of the time-averaged data, using the CSD algorithm described by Junghöfer et al. (1997) and λ = 0.2, as recommended for dense-array EEG montages. The CSD time series values were then transformed into the frequency domain using a Fast Fourier Transformation on a time interval between 600 and 3900 ms after stimulus onset. The first 600 ms after stimulus onset were omitted to reduce the impact of initial non-stationary components of the ssVEP on the power spectrum (see Fig. 2A; Miskovic & Keil, 2014). In a next step, we extracted the signal-to-noise ratio (SNR) for the driving frequency of 7.5 Hz by dividing the power of the frequency of interest by the mean of the spectral power at six adjacent frequency bins, leaving out the two immediate neighbors. Additionally, given that the frequency spectrum analysis (Fig. 2B) revealed a pronounced ssVEP response at the second harmonic (15 Hz), we also extracted the SNR at 15 Hz for further exploratory analyses. For statistical analysis, the ssVEP activity was pooled across sensor Oz and seven neighboring electrodes (see Fig. 2C; EGI sensors 70, 71, 72, 74, 75, 76, 82, 83; Stegmann et al., 2020; Wieser & Keil, 2014).

### 2.5 Statistical analyses

Mean differences in ssVEP 7.5 Hz SNRs, pupil responses, and US-expectancy ratings during the context phase were analyzed using repeated-measures ANOVAs with the within-subject factors Cue (eight levels: -45°, +15°, +25°, +35°, +45°/CS+, +55°, +65°, +75°) and Context (two levels: CTX_a_ vs. CTX_n_). Cue differences during acquisition were tested using the same model, replacing the factor Context with the factor Time (two levels: 1^st^ vs. 2^nd^ half of acquisition) to account for the temporal dynamics of learning. Note that for US-expectancy, the factor Time was omitted, as these ratings were collected only once, after the whole acquisition phase. Since the visual flicker elicited a pronounced ssVEP at the second harmonic (15 Hz, see Fig. 2B) of the driving frequency, all statistical analyses were repeated in an exploratory manner for the harmonic frequency response. All analyses were conducted in the R software environment (version 4.2.2.; R Development Core Team, 2021), using the *afex*-package for ANOVAs (Singmann et al., 2020; version 1.2-1), and a significance level of α = .05. Confidence intervals (95%) for Cohen’s *d* and partial eta-squared ($\eta_{p}^{2}$) were calculated with the *MBESS* (Kelley, 2020; version 4.9.2) and *apa* (Gromer, 2020; version 0.3.3) packages.

To follow up on the results of the ssVEP amplitudes during the context phase, and to directly compare the fits of a lateral inhibition (sharpening) versus generalization pattern, we calculated Bayesian linear models. For this analysis, we pre-specified weight vectors, that represented the expected generalization profiles under a sharpening or a generalization model, respectively. These weight vectors were then entered into a linear regression as predictors for the factor Cue. For the lateral inhibition and generalization pattern, the same weight vectors were used as in the study by McTeague et al. (2015). The lateral inhibition pattern was expressed as the difference of two Gaussians (weights: +0.5, -1, -2, +5, -2, -1, +0.5 for + 15°, +25°, +35°, +45°/CS+, +55°, +65°, +75°). For the cue generalization pattern, a quadratic trend was used (weights: -3, +0.5, +1.5, +2, +1.5, +0.5, -3). In each model, the factor Context was entered as an additional predictor variable and subjects were entered as random intercepts to the model. To analyze the topographies of the model fits, transitive Bayes factors (BFs) for the main effects of cue and context were then calculated for each predictor weight model, EEG sensor, and frequency (7.5 Hz and 15 Hz) by comparing each candidate model against null model (assuming no differences between cue and/or context conditions). The resulting BFs can be interpreted as measures of evidence in favor of the sharpening or generalization model compared to the null model. Higher BFs indicate stronger support for the respective model, suggesting that the corresponding pattern (sharpening, generalization, main effect of context) better explains the observed ssVEP amplitude distribution across conditions and electrodes. Bayesian analyses were conducted in R, using the package ‘BayesFactor’ (version 0.9.12-4.4) and default JZS-priors (Rouder & Morey, 2012).

## 3 Results

### 3.1 Acquisition

**Fig. 3.**
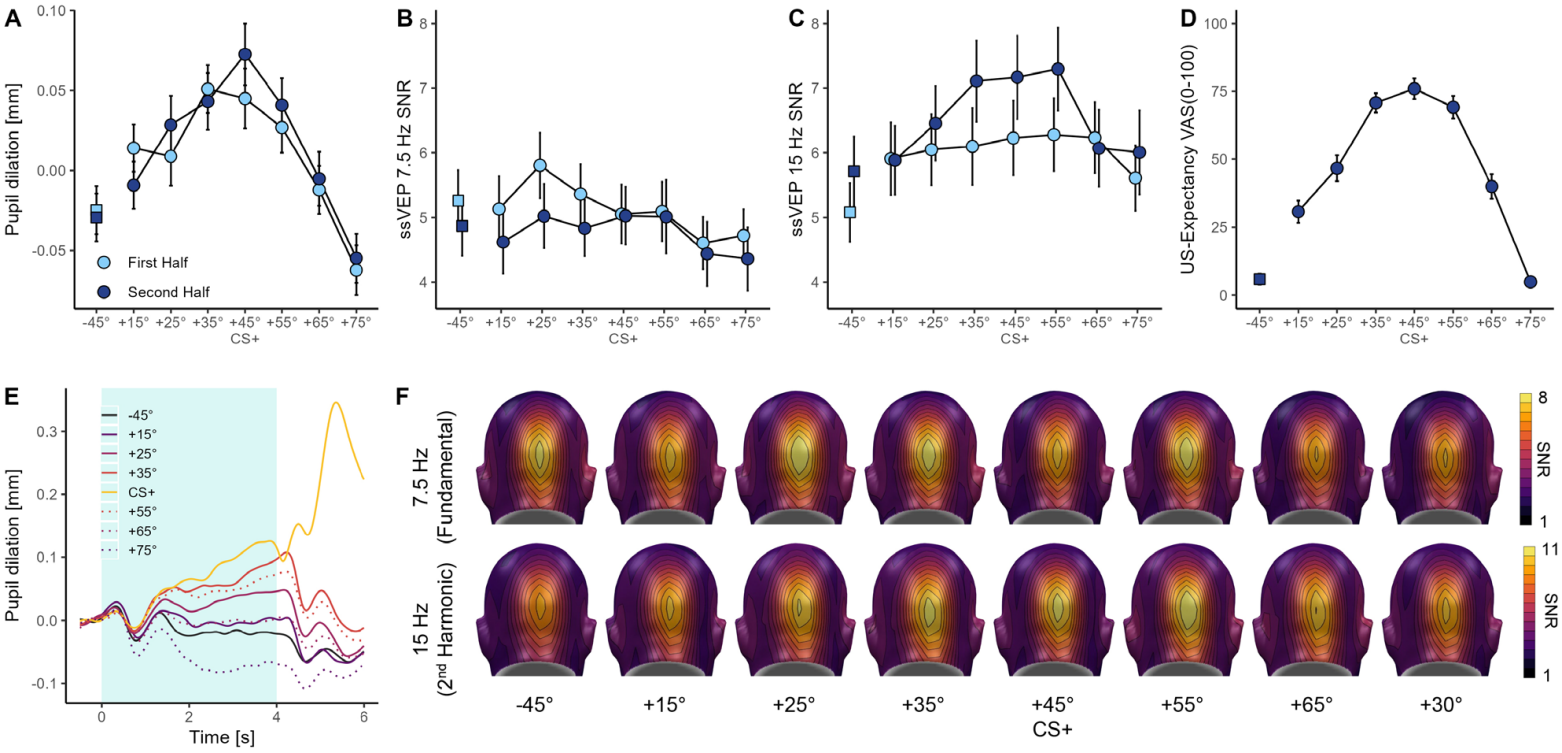
Results of the acquisition phase. (A) Mean (± SEM) pupil responses, (B) 7.5 Hz ssVEP-SNR responses, and (C) 15 Hz ssVEP-SNR responses to the conditioned stimuli during the first (light blue) and second half (dark blue) of the acquisition phase. (D) US-expectancy ratings, collected only at the end of the acquisition phase. (E) Average changes in pupil dilation in response to cue onset across the entire acquisition phase; the shaded area indicates the cue presentation window. In CS+ trials, an electrical stimulus was delivered at cue offset (4 s). (F) Averaged scalp topographies of the 7.5 Hz and 15 Hz ssVEP-SNRs across the whole acquisition phase.

For US-expectancy ratings after the acquisition phase, there was a main effect of cue, *F*(4.09, 208.81) = 63.22, *p* < .001, *η_p_^2^* = .56, *CI*_95_ = [.48; .60], A post-hoc t-test revealed a significant difference between the CS+ and the opposite (CS_-45°_) orientation, *t*(51) = 15.04, *p* < .001, *d* = 2.09, *CI*_95_ = [1.60; 2.57]. Furthermore, US-expectancy ratings were decreasing with increasing dissimilarity to the CS+ (see Fig. 3D), suggesting successful threat learning and generalization.

A similar pattern emerged for pupil responses, where the ANOVA showed a main effect of cue, *F*(4.84, 247.01) = 13.53, *p* < .001, *η_p_^2^* = .21, *CI*_95_ = [.13; .27], but no differences between the first and second half of acquisition, Time: *F*(1, 51) = .32, *p* = .573, *η_p_^2^* < .01, *CI*_95_ = [.00; .11], Cue x Time: *F*(7, 357) = .90, *p* = .504, *η_p_^2^* = .02, *CI*_95_ = [.00; .03]. As for the ratings, the post-hoc t-test between the CS+ and the opposite (CS_-45°_) orientation during the second half of the acquisition revealed a significant difference, *t*(51) = 4.18, *p* < .001, *d* = .58, *CI*_95_ = [.28; .87], suggesting successful fear learning, while pupillary responses were decreasing with decreasing similarity with the CS+ orientation (Fig. 3A and 3E).

The ANOVA for the fundamental (7.5 Hz) frequency of the ssVEP response revealed a significant difference between cues, *F*(5.38, 215.07) = 2.96, *p* = .011, *η_p_^2^* = .07, *CI*_95_ = [.01; .11] and generally higher SNRs during the first compared to the second half of the acquisition phase, *F*(1, 40) = 5.63, *p* = .023, *η_p_^2^* = .12, *CI*_95_ = [.00; .31]. The Cue x Time interaction was not significant, *F*(5.05, 202.09) = .66, *p* = .656, *η_p_^2^* = .02, *CI*_95_ = [.00; .03]. However, the results did not follow any of the expected generalization or sharpening patterns (see Fig. 3B and 3F). Consistently, the post-hoc t-test revealed no significant difference between CS+ and CS_-45°_ during the second half of the acquisition phase, *t*(40) = .05, *p* = .958, *d* < .01, *CI*_95_ = [-.31; .30].

In contrast, the exploratory analysis of the second harmonic (15 Hz) of the ssVEP response revealed a significant main effect of cue, *F*(5.31, 212.29) = 7.45, *p* < .001, *η_p_^2^* = .16, *CI*_95_ = [.07; .22], and Time, *F*(1, 40) = 8.43, *p* = .006, *η_p_^2^* = .17, *CI*_95_ = [.02; .37]. The Cue x Time interaction was marginally significant, *F*(5.22, 208.65) = 2.06, *p* = .069, *η_p_^2^* = .05, *CI*_95_ = [.00; .08], indicating slightly stronger threat discrimination in the second half of acquisition (see Fig. 3C). Accordingly, the contrast between CS+ and the CS_-45°_ orientation during the second half of acquisition was significant, *t*(40) = 3.55, *p* < .001, *d* = .55, *CI*_95_ = [.22; .88], and responses decreased with increasing dissimilarity to the CS+, thus indicating successful threat generalization.

### 3.2 Context phase

#### 3.2.1 US-expectancy ratings

**Fig. 4.**
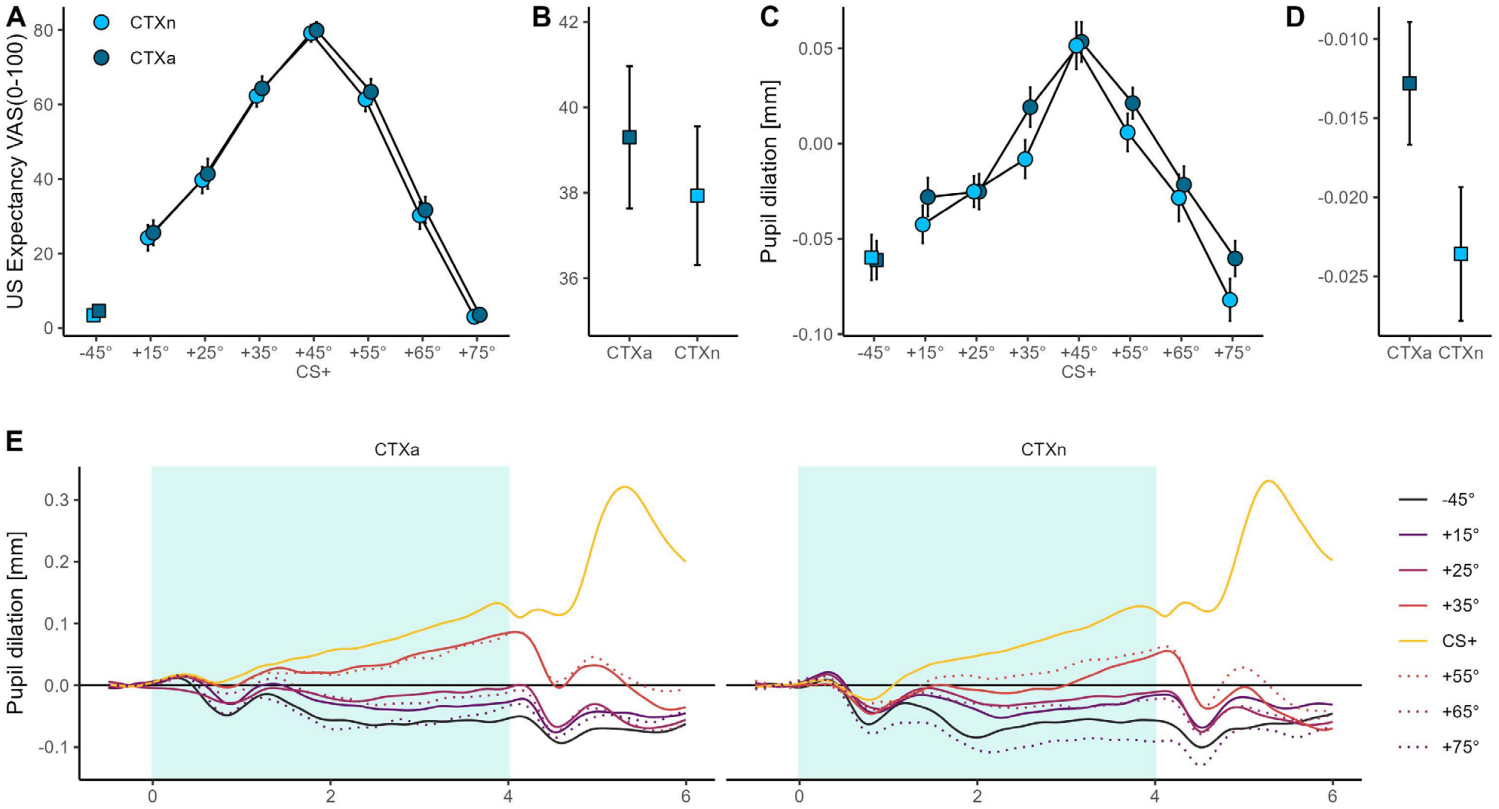
Results for pupil responses and US-expectancy ratings in the context phase. Mean (± *SEM)* US-expectancy ratings (A) and pupil responses (C) to the conditioned stimuli as a function of context, as well as mean (± *SEM)* US-expectancy ratings (B) and pupil responses (D) averaged across all cues to illustrate the main effect of context. (E) Average changes in pupil dilation in response to the cue onset during the aversive (left) and neutral context (right). The shaded area indicates the time window of cue presentation. In CS+ trials, an electrical stimulus was presented at the offset of the cue at 4 s.

The ANOVA on US-expectancy ratings revealed a main effect of cue, *F*(3.43, 174.79) = 149.20, *p* < .001, *η_p_^2^* = .75, *CI*_95_ = [.70; .77], and context, *F*(1, 51) = 4.49, *p* = .039, *η_p_^2^* = .08, *CI*_95_ = [.00; .24], suggesting a robust generalization gradient and that participants expected the electrical stimulus more strongly when the central cues were presented in the aversive context (see Fig. 4A). The interaction was not significant, *F*(4.82, 254.61) = .10, *p* *=* .991, *η_p_^2^* = .01, *CI*_95_ = [.00; .00].

#### 3.2.2 Pupil responses

During the context phase, pupil responses to the central cues revealed significant main effects of cue, *F*(4.24, 216.13) = 27.27, *p* < .001, *η_p_^2^* = .35, *CI*_95_ = [.26; .41], and context, *F*(1, 51) = 8.69, *p* = .005, *η_p_^2^* = .15, *CI*_95_ = [.01; .32] indicating overall stronger responses to the central cues in the aversive compared to the neutral context, alongside the expected generalization gradient (see Fig. 4B and C). The interaction between cue and context was not significant, *F*(7, 357) = .89, *p* = .515, *η_p_^2^* = .02, *CI*_95_ = [.00; .03].

#### 3.2.3 Visuocortical responses

**Fig. 5.**
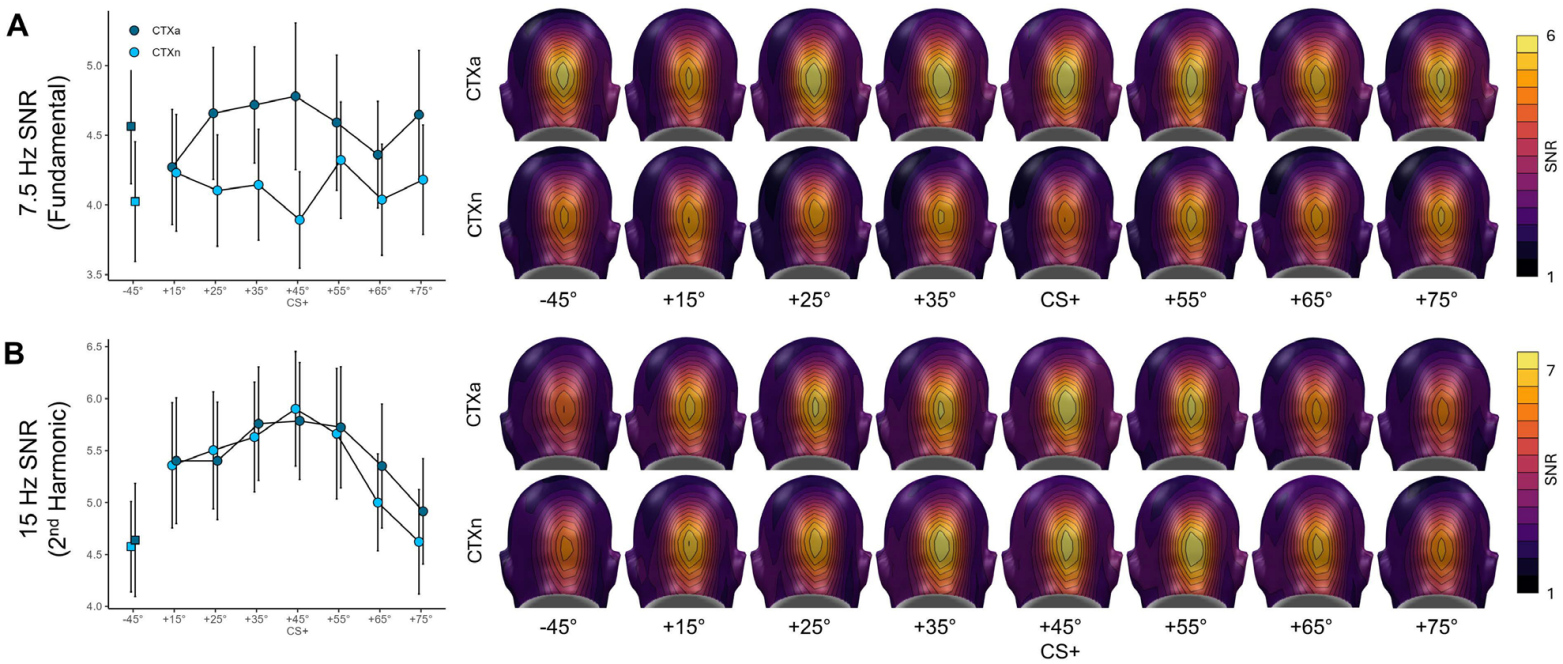
Results for visuocortical responses of the context phase. Mean (± *SEM)* 7.5 Hz (A) and 15 Hz (B) ssVEP-SNR responses to the conditioned stimuli as a function of context and their respective topographies.

Analysis of the ssVEP-SNRs during the context phase revealed substantial differences between the fundamental (7.5 Hz) and harmonic (15 Hz) frequency responses (see Fig. 5). For the fundamental frequency response, there was a main effect of context, *F*(1, 47) = 10.96, *p* = .002, *η_p_^2^* = .19, *CI*_95_ = [.03; .37], without a main effect of cue, *F*(4.94, 232.12) = .42, *p* = .830, *η_p_^2^* = .01, *CI*_95_ = [.00; .01]. Conversely, for the harmonic frequency response, a main effect of cue was found, *F*(4.83, 227.18) = 8.44, *p* < .001, *η_p_^2^* = .15, *CI*_95_ = [.07; .21], but no main effect of context, *F*(1, 47) = .40, *p* = .530, *η_p_^2^* < .01, *CI*_95_ = [.00; .12]. Indeed, post-hoc t-tests for the fundamental frequency showed that the difference between CS+ and CS_-45°_ was not significant in the aversive, *t*(47) = 0.63, *p* = .534, *d* = .09, *CI*_95_ = [-.19; .37], or neutral context, *t*(47) = .44, *p* = .661, *d* = .06, *CI*_95_ = [-.22; .35], whereas for the harmonic frequency the same tests between CS+ and CS_-45°_ was significant in both the aversive, *t*(47) = 3.90, *p* < .001, *d* = .56, *CI*_95_ = [.26; .87], and neutral context, *t*(47) = 4.00, *p* < .001, *d* = .58, *CI*_95_ = [.27; .88]. The interaction was neither significant for the 7.5 Hz, *F*(7, 329) = 1.19, *p* *=* .309, *η_p_^2^* = .02, *CI*_95_ = [.00; .04], nor the 15 Hz frequency response, *F*(5.08, 238.76) = .35, *p* *=* .887, *η_p_^2^* < .01, *CI*_95_ = [.00; .01].

**Fig. 6.**
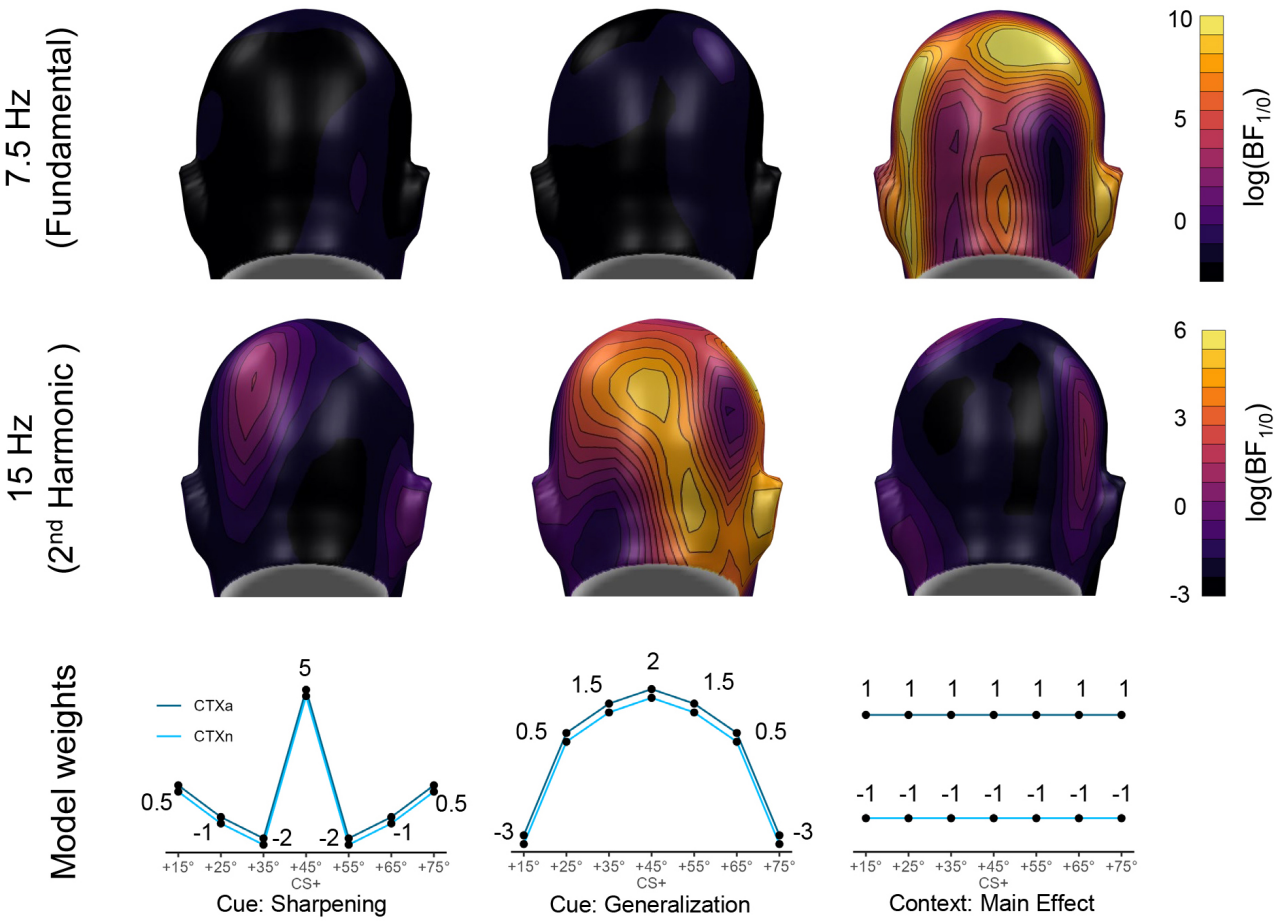
Topographical distributions of the Bayes Factors for comparing the main effects of cue and context to the null model for each contrast using Bayesian linear mixed models *(SNR ~ weights* *x* *Cue* *+* *Context).* Weights used for the contrasts are displayed at the bottom row and represent the expected generalization profiles under a sharpening or generalization model, respectively. For the sharpening and generalization model fits, the same cue weights were chosen for both contexts. To illustrate the main effect of context, the weights were the same for all cues but differed between contexts. Natural log-transformed BFs are illustrated, so that positive values display support for the full effect model while negative values display support for the null model. Higher BFs indicate stronger support for the respective model, suggesting that the corresponding pattern (sharpening, generalization, main effect of context) better explains the observed ssVEP SNR than the null model (assuming no differences between cue or context conditions).

To follow up on these findings, we used Bayesian linear mixed models to directly compare the topographies of a sharpening and generalization model of the main effect of cue and the topographies of the main effect of context between the fundamental and harmonic frequency response (see Fig. 6). Indeed, the results demonstrated strong evidence (BF_M/0_ > 1000) for a main effect of context over parieto-occipital sensors for the fundamental, but not the harmonic frequency, whereas the cue generalization pattern was only evident (BF_M/0_ > 1000) over parieto-occipital sensors for the harmonic, but not the fundamental frequency. Evidence for a sharpening model for the main effect of cue could neither be found for the fundamental nor the harmonic frequency.

## 4 Discussion

The aim of the current study was to investigate how generalized fear responses are influenced by aversive contextual information. Building on previous research (Stegmann et al., 2022, 2024), we hypothesized that visuocortical and somato-visceral responses as well as subjective ratings would be elevated in aversive compared to neutral contexts. Furthermore, we tested to what extent the electrocortical correlates of sensory processing follow an additive model, characterized by a general pattern of threat generalization with overall enhanced responses in the aversive compared to the neutral context; or an interactive model, characterized by a sharper or broader generalization gradient in the aversive relative to the neutral context.

For pupil responses and US-expectancy ratings, which served as indices of sympathetic nervous system activity and cognitive threat expectations, respectively, the results during the acquisition and context phases exhibited the expected generalization gradient in response to the central cues, with the strongest responses to the CS+ and gradually decreasing responses as similarity to the CS+ diminished in both directions along the orientation continuum. This pattern substantiates a growing body of aversive generalization literature, demonstrating generalization of conditioned threat in subjective (Stegmann, Schiele, et al., 2019) and somato-visceral measures, such as skin conductance (Dunsmoor et al., 2009), pupillometry (Reutter & Gamer, 2023), startle-potentiation (Lissek et al., 2008), and heart rate (Ahrens et al., 2016). In addition, our results for the context phase revealed generally heightened US-expectancy and pupil responses in aversive compared to neutral contexts, regardless of the orientation of the central cue. Although the effect sizes for the impact of aversive contexts on conditioned fear responses were small, they were consistent with previous findings (Stegmann et al., 2022, 2024), indicating a relatively robust phenomenon. From an evolutionary perspective, both mechanisms—threat generalization and heightened responses in aversive situations—are considered adaptive (Dunsmoor & Paz, 2015; Fanselow, 2018) but although both serve the goal of enhanced survival in situations of uncertain threat, they achieve this through different means. Generalization relies on prior learning experiences, transferring conditioned responses from a stimulus that has originally been associated with threat to a novel yet similar stimulus. As a result, the characteristics of the generalized response closely resemble the original threat response, though it becomes less intense as the similarity decreases. Importantly, threat generalization conserves cognitive and physiological resources by allowing organisms to respond to potential dangers without requiring specific learning for each threat, thereby enabling more efficient resource allocation in situations with an elevated risk of harm. In order to benefit from aversive generalization mechanisms, however, organisms are still required to initially identify a specific, potentially threatening stimulus. In contrast, aversive contexts prepare the organism for an upcoming danger even before an actual threat is identified. It has been argued that these situations of diffuse threat induce heightened vigilance to improve the detection of potential threats, which is accompanied by a sustained state of moderate defense system activation, preparing the organism for defensive action once an actual threat is recognized (Blanchard & Blanchard, 1989; Fanselow, 2018; Lang et al., 2000). Thus, contextual information is another factor in optimizing defensive behavior (Mobbs et al., 2015), as a sustained state of moderate defense system activation may strike the ideal balance between minimizing the cost of defensive resources and enhancing survival chances in situations of potential danger.

Until now, however, it remained rather unclear how these mechanisms interact, specifically how aversive contexts influence threat generalization. In the current study, the absence of significant interaction effects for US-expectancy ratings and pupil responses provides unanimous evidence for an additive rather than an interactive model of threat generalization and contextual modulation of conditioned fear responses. Interactions between threat generalization and aversive contexts would have been evident in a difference in the shape of the generalization gradient between the aversive and neutral contexts. For example, heightened vigilance in the aversive context could have led to better discrimination between central cue orientations, resulting in a sharper generalization gradient. Conversely, a sustained activation of the defense system might have elicited a generalized ‘better-safe-than-sorry” strategy, leading to overgeneralization of conditioned threat in the aversive context and thereby producing a broader generalization gradient. Instead, our results for US-expectancy ratings and pupil responses showed generalization gradients with a similar shape that only differed by an increased vertical offset in the aversive compared to the neutral context. This pattern is in line with a linear combination between effects of aversive contexts and threat generalization (Grillon & Charney, 2011; Stegmann et al., 2022, 2024). Additive combinations of different threat-processing mechanisms suggest relatively independent neural functions, enabling the brain to dynamically adjust its response based on the specific environmental demands.

This independence was even more evident in the response pattern for visuocortical activity: the initial analysis of the fundamental driving frequency (7.5 Hz) showed no specific response pattern to the central cues, neither widening nor sharpening. During the context phase, however, the 7.5 Hz frequency was strongly modulated by the context, with overall higher responses to the central cues in the aversive compared to the neutral context. Furthermore, since the grand average indicated a strong SNR at the 2^nd^ harmonic frequency (15 Hz), we conducted an exploratory analysis of changes in the 15 Hz frequency in response to the flickering central cues, which revealed a generalization pattern similar to the findings from the behavioral and pupil responses. In addition, the 15 Hz frequency did not appear to be sensitive to contextual information, as the generalization gradients fully overlapped for aversive and neutral contexts. Together, these findings suggest that the fundamental frequency was modulated by the contexts, while the 2^nd^ harmonic frequency was modulated by the cues. To further clarify the dissociation between cue and context reactivity in the fundamental and harmonic frequencies, we compared the fits and topographies of these effects using Bayesian-linear-models. This analysis revealed that both effects are evident at sensors over parieto-occipital areas, with context effects in the fundamental frequency showing a somewhat more widespread activation pattern than cue effects in the harmonic frequency. This finding aligns with previous studies investigating visuocortical responses to conditioned contexts, which similarly reported broader activity patterns for context effects compared to cue effects (Stegmann, Reicherts, et al., 2019; Wieser et al., 2016). Importantly, context-related effects were also observed at occipital sensors, which served as the basis for statistical analysis in the present study. In addition, the Bayesian analysis confirmed that the 7.5 Hz frequency was sensitive to contextual information but not the central cues, while the 15 Hz frequency was modulated by the central cues but not by the contexts. In interpreting these findings, it is important to consider that the current task imposed two potentially conflicting demands on the attentional system: On the one hand, central cues indicating imminent threat require selective attention (Richards et al., 2014), which is known to produce stronger visuocortical activity for threat-related cues compared to safety cues (Miskovic & Keil, 2012). On the other hand, aversive contexts induce a general state of hypervigilance aimed at improving threat detection (Richards et al., 2014). As both attentional states are motivationally relevant, it seems crucial to have developed a mechanism for maintaining both states simultaneously, allowing the organism to focus on immediate threats while remaining aware of other potential dangers, rather than neglecting situational risks once a threat has been identified. To reconcile these competing demands, our results suggest that different harmonics of the visuocortical response might code different aspects of attentional regulation. Indeed, Kim et al. (2011) demonstrated that under certain conditions, fundamental and harmonic frequencies exhibit different sensitivities to top-down modulations during a voluntary attentional task, due to the involvement of distinct neuronal populations in generating the fundamental and harmonic frequency. Because of their receptive-field properties and sensitivity to specific luminance polarities, simple cells produce a frequency-following neural response when presented with a flickered stimulus, while complex cells generate a frequency-doubling response (Benucci et al., 2007; Kim et al., 2011). Unfortunately, the functional significance of different ssVEP harmonics remains largely understudied. In addition, the segregation of frequency-following and frequency-doubling neuronal populations in generating fundamental and harmonic frequencies is not straightforward and depends on the type of flicker mode used (Kim et al., 2011). In the current study, we used an “on-off” flicker, where the fundamental frequency is produced by an interplay of frequency-following and frequency-doubling neurons, while the second harmonic is generated exclusively by frequency-doubling neurons. Assuming a harmonic-based segregation of attentional mechanisms, our results suggest that frequency-doubling neurons are more strongly influenced by visuocortical tuning in response to aversive cue learning, that is, altering their preference for threat-signaling stimulus properties (Antov et al., 2020; Friedl & Keil, 2020, 2021; McTeague et al., 2015; Stegmann et al., 2020). In contrast, frequency-following neurons might be more susceptible to contextual modulations, reflecting a generally heightened neural reactivity of simple cells during states of hypervigilance, induced by relatively slower feedback projections from frontal areas (Alvarez et al., 2011; Langner & Eickhoff, 2013). Taking into account that the current interpretations are based on exploratory post-hoc analyses, it seems important for future studies to further substantiate this hypothesis of harmonic-based segregation of visuocortical mechanisms related to competing attentional demands. Such research should consider implementing a “light-dark” flicker (i.e., alternating phases of lighter and darker stimulation relative to the background) since this approach would allow for a complete dissociation of frequency-following and frequency-doubling neuronal responses into the fundamental and second harmonics of the ssVEP (Kim et al., 2011).

Another interesting consideration is that the increased 7.5 Hz ssVEP responses during aversive contexts may not solely reflect arousal-driven modulation of frequency-following neural populations. Rather, the spatial overlap of the central cues with the background images could also contribute directly to the flicker response. Although the background images were static in the current study and varied between blocks, the rhythmic offset of the flickering Gabor stimuli may have acted as quasi-onsets of the background content, potentially amplifying neural responses at the driving frequency. Consistent with this idea, prior studies have shown that flickering aversive images evoke stronger ssVEP amplitudes than neutral ones (Keil et al., 2003). Nevertheless, the modulation of the 15 Hz frequency by the central cues suggests that context and cue-related processes interact in a more complex manner, which warrants further investigation.

It is important to note that in our previous study (Stegmann et al., 2022), which employed a differential fear conditioning task in front of aversive and neutral contexts, we observed a significant main effect of cue and a marginally significant main effect of context on the 7.5 Hz fundamental frequency of the ssVEP. The presence of both effects in the fundamental frequency could be attributed to the involvement of neural populations responsible for frequency-following and frequency-doubling as stated above. In contrast to the eight orientations used in the current study, however, only two differential cues were used, which might have been insufficient to detect a harmonic-based segregation of attentional mechanisms.

In a recent study, Aslanidou et al. (2025) also investigated the effects of threatening and safe contexts on generalization processes using a fear generalization paradigm. In their study, the CS+ was paired with an aversive US in the threatening context, but not in the safe context. Although participants showed higher US-expectancy ratings in the threatening context, they did not exhibit stronger cue generalization for ssVEP amplitudes or skin conductance responses compared to the safe context. Two key differences between their study and the present research are noteworthy. First, Aslanidou et al. did not employ an orthogonal design; instead, the meanings of the cues were context-dependent, with the CS+ predicting a US exclusively in the threatening context and not in the safe context. Second, the present study uses naturalistic, inherently threatening pictures as contexts, whereas Aslanidou et al. relied on conditioned, geometric symbols as contextual cues. This notion aligns with the results of our previous study (Stegmann et al., 2024), where we only observed effects of inherently aversive contexts, but not of conditioned aversive contexts on central cue processing. Thus, more research is necessary to explore the impact of different kinds of context on threat responses.

Contrary to our expectations, we did not observe a pattern of sharpened re-tuning in response to the conditioned central cues, as seen in previous threat generalization tasks (Antov et al., 2020; McTeague et al., 2015; Stegmann et al., 2020). One possible explanation is that those studies employed a higher flicker frequency (12–15 Hz) compared to the 7.5 Hz driving frequency used in the current study. It remains unclear how emotional attention influences different frequencies, although varying effects for different frequencies using the same task have been reported (Bekhtereva et al., 2018, 2019). Furthermore, McTeague et al. (2015) and Antov et al. (2020) used a ‘counterphase’ flicker, where lighter and darker portions of the stimulus were presented simultaneously but modulated by contrast reversal at each flicker cycle. In counterphase flicker, the responses of frequency-following neurons are theoretically cancelled out, allowing only the responses of frequency-doubling neurons, measured as the second harmonic of a full reversal cycle, to be observed (Kim et al., 2011). This could explain why the authors detected effects of aversive cue conditioning at this frequency.

It is important to acknowledge that we cannot entirely rule out the possibility that participants perceived the combination of central and context cues as a single compound stimulus, rather than as separate foreground and background elements. However, we incorporated several design features to minimize the formation of entirely new cue–context compound associations. These included the sequential (rather than simultaneous) onset of context and cue stimuli, as well as the continuous presence of the context throughout each block. Additionally, each context picture was presented only once during the experiment, encouraging participants to rely on previously learned CS–US contingencies rather than forming new cue–context associations.

Taken together, the current study provides further evidence for an additive model of conditioned fear responses to imminent threat-related cues in aversive contexts (Stegmann et al., 2022, 2024). By orthogonally combining fear generalization with aversive vs. neutral contexts, we observed the expected generalization gradients for US-expectancy and pupil dilation. These gradients were characterized by elevated responses to the threat-associated central cue and decreasing responses with diminishing similarity, with overall heightened gradients in aversive compared to neutral contexts. Additionally, we found that for visuocortical activity this pattern was separated into different frequencies: the fundamental frequency was sensitive to contextual modulations, while the second harmonic frequency of the ssVEP was sensitive to the conditioned central cues. These findings offer novel insights into adaptive defensive behavior in more complex situations and suggest potential mechanisms for resolving competing attentional demands through harmonic-based segregation among frequency-following and frequency-doubling neural populations.

## Data Availability

All data and code that support the findings of this study are openly available at https://osf.io/jhvm4/.
